# Effect of bilateral uterine artery ligation on blood loss during total laparoscopic hysterectomy

**DOI:** 10.3389/fmed.2025.1577904

**Published:** 2025-05-02

**Authors:** Ahmed El-Minawi, Hossam El-shenoufy, Ahmed Hussein, Hesham El-fazary, Mostafa M. Bahaa, Alaa A. Alsharif, Hayam Ali AlRasheed, Mostafa Eldardiry

**Affiliations:** ^1^Department of Obstetrics and Gynecology, Faculty of Medicine, Cairo University, Giza, Egypt; ^2^Department of Obstetrics and Gynecology, Faculty of Medicine, Alexandria University, Alexandria, Egypt; ^3^Pharmacy Practice Department, Faculty of Pharmacy, Horus University, New Damietta, Egypt; ^4^Department of Pharmacy Practice, College of Pharmacy, Princess Nourah Bint Addulrahman University, Riyadh, Saudi Arabia; ^5^Department of Obstetrics and Gynecology, Faculty of Medicine, Kafr Alsheikh University, Kafr el-Sheikh, Egypt

**Keywords:** total laparoscopic hysterectomy, uterine artery ligation, blood loss, operative time, surgical complications, postoperative recovery

## Abstract

**Background:**

Hysterectomy is a common major gynecological surgery. Total laparoscopic hysterectomy (TLH) has become a preferred method over traditional approaches due to its minimally invasive nature and reduced postoperative complications.

**Aim:**

This study aimed to compare conventional total laparoscopic hysterectomy (CTLH) with TLH involving bilateral uterine artery ligation (BUAL) at its origin, specifically evaluating blood loss and perioperative outcomes.

**Methods:**

In this prospective randomized study conducted at Cairo University Hospital, 60 female patients undergoing TLH for benign uterine conditions were randomized. Group 1 (BUAL) involved bilateral uterine artery ligation at its origin, and Group 2 (CTLH) followed conventional TLH techniques. Preoperative assessments included comprehensive history, physical examinations, and relevant laboratory tests. Outcomes measured were intraoperative blood loss, operative time (from insufflation to skin suturing), intraoperative and postoperative complications, postoperative analgesic needs, and hospital stay.

**Results:**

Both groups were demographically similar. The BUAL group experienced significantly lower blood loss (103.7 ± 23.27 mL) compared to the CTLH group (128.7 ± 42.57 mL) (*p* < 0.05). However, the mean operative time was slightly longer in the BUAL group (169.33 ± 15.85 min) than in the CTLH group (160.50 ± 19.75 min). No major surgical complications or blood transfusions were reported in either group. The posterior approach to uterine artery ligation in the BUAL group was more time-efficient.

**Conclusion:**

Securing the uterine arteries at their origin during TLH reduces blood loss and provides a feasible alternative to conventional methods, despite a slightly longer operative time. Enhanced surgical expertise correlates with reduced operative duration and improved outcomes.

## 1 Introduction

Following cesarean section, hysterectomy is the second most common major gynecological surgery, with approximately 600,000 procedures performed annually in the USA (1). Since Reich et al. first reported total laparoscopic hysterectomy (TLH) in 1989, numerous studies have confirmed its feasibility and reproducibility (2, 3). Evidence increasingly supports TLH over vaginal hysterectomy (VH) and total abdominal hysterectomy (TAH) for benign gynecological diseases (4). The development and rapid advancement of laparoscopic instruments and techniques have made it possible to safely and successfully complete complex procedures using minimally invasive approaches. Women with a higher body mass index (BMI) or those requiring complex surgery benefit from reduced postoperative complications with laparoscopic procedures (5).

Numerous studies have shown the benefits of operative laparoscopy over laparotomy, with the risk of complications remaining manageable. These benefits include significant reductions in the time of hospital stay, estimated blood losses, the need for analgesics, and postoperative pain. Laparoscopic hysterectomy has been found to improve recovery and quality of life (6–12). However, it significantly prolongs surgical time, as reported in randomized and observational studies (13–16).

The uterine and ovarian arteries provide the majority of the uterus’s circulatory supply. Following uterine artery ligation, temporary uterine ischemia occurs because the majority of blood reaches the uterus through the uterine arteries (17). One effective way to stop the blood supply to the uterus is to ligate both uterine vessels (18). The uterine vascular pedicle must be secured as the primary step in a hysterectomy (19). Myometrial blood clotting and the myometrium become hypoxic shortly after occlusion (20). Depending on the size and location of myomas, enlarged uteri may have limited accessibility to the uterine vascular pedicles and may be linked to a higher risk of problems such as ureteral injury and bleeding. Prior ligation and dissection of the uterine artery at its origin reduces the risk of ureteral injuries and the blood supply (19, 21).

However, TLH carries a specific risk of complications, notably a 2.6-fold higher risk of urinary tract injury (22). Despite these risks, operative laparoscopy offers advantages over laparotomy, including reduced blood loss, decreased analgesic requirements, reduced postoperative pain, shorter hospital stays, and improved recovery and quality of life (23). However, it extends surgical time, as documented in both randomized and observational studies (13, 14).

In light of these observations, the goal of this prospective clinical randomized experiment was to compare conventional TLH with bilateral uterine artery ligation at its origin, aiming to decrease intraoperative blood loss and perioperative complications with total laparoscopic hysterectomy.

## 2 Patients and methods

The study was conducted at Cairo University Hospital’s laparoscopic surgery unit from February 2020 to February 2022. This study was approved by the National Research Ethical Committee of Cairo University. The trial population consisted of 60 female patients undergoing total laparoscopic hysterectomy (TLH) for benign uterine conditions. Thirty women underwent TLH with ligation of the uterine artery at the cervix, and 30 women underwent TLH with bilateral ligation of the uterine artery at its origin from the internal iliac artery.

**Figure 1:**
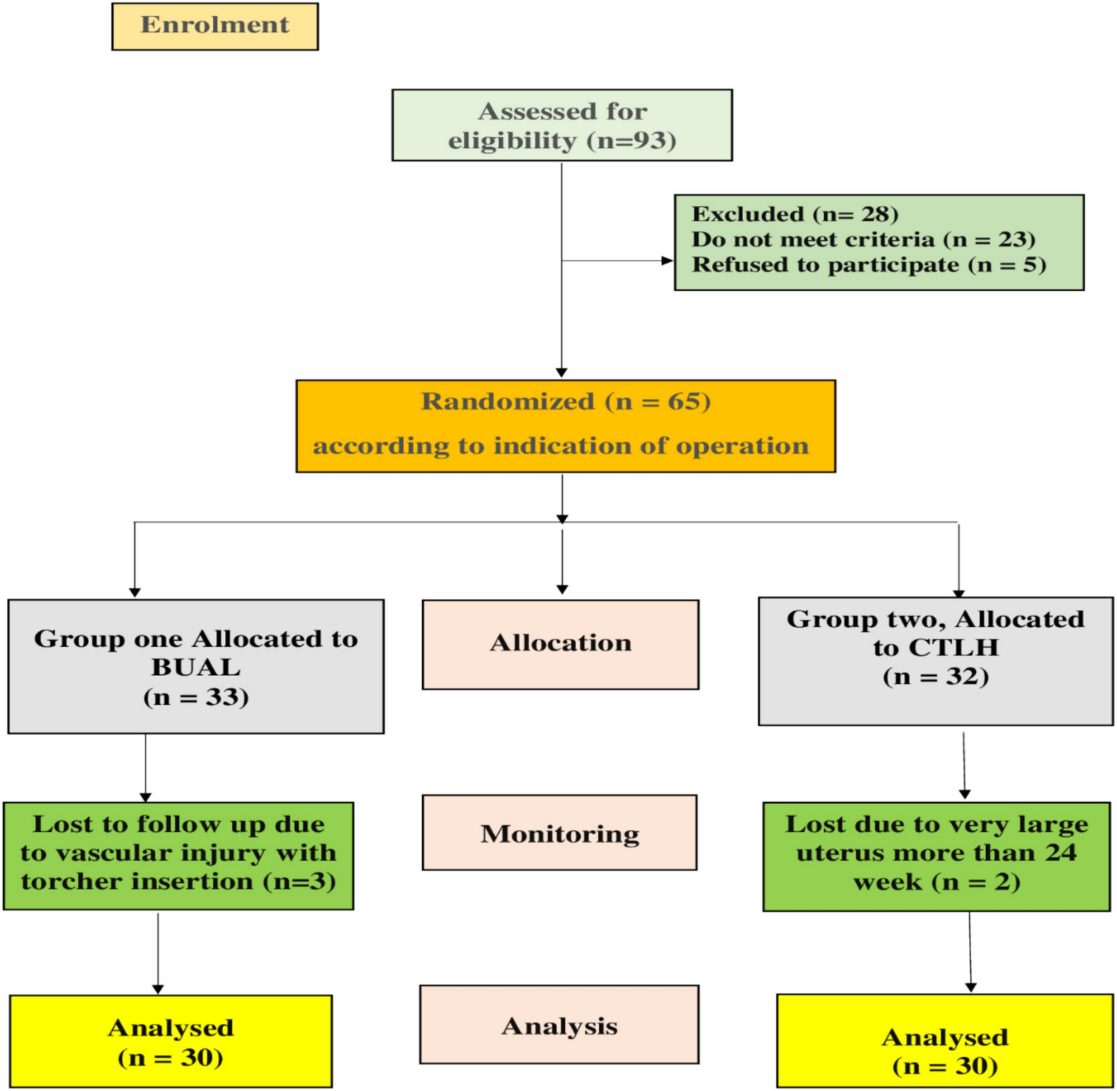
CONSORT flowchart displaying the flow of participants during the study.

Patients recruited for this study were those presenting to the Department of Obstetrics & Gynecology for hysterectomy consultation. Informed consent was obtained from all patients, and they had the right to withdraw from the trial. A computer-generated random number was used in the randomization process. A total of 60 patients who fulfilled the criteria and provided written informed consent were randomly assigned to one of the two groups according to the CONSORT diagram (Figure 1).

### 2.1 Inclusion criteria

Patients aged between 35 and 60 years, patients presenting with menometrorrhagia unresponsive to medical treatment, and uterine pathology such as adenomyosis or multiple fibroids were included in this study.

### 2.2 Exclusion criteria

Patients were excluded if they had medical conditions preventing pneumoperitoneum or medical conditions hindering proper ventilation during general anesthesia. Furthermore, patients diagnosed with endometrial carcinoma and patients with uterine size larger than 24 weeks were excluded. Patients with excessive adhesions that preclude access to the uterine arteries were not enrolled in the study.

### 2.3 Study design

Patients underwent comprehensive history taking, general, abdominal, and vaginal examinations, ultrasound investigations, and laboratory tests including complete blood count (CBC), prothrombin time (PT), partial thromboplastin time (PTT), international normalization ratio (INR), random blood sugar (RBS), liver, and renal functions.

Patients were randomly allocated to each group for age, body mass index (BMI), number of previous operations, size of the uterus, and type of uterine pathology.

#### 2.3.1 Group 1 (BTLH with bilateral uterine artery ligation from its origin)

The round ligament close to the pelvic side wall is first coagulated and separated before the procedure is applied. A further incision is then made in the peritoneum. The bladder fold is pulled downward by opening the anterior leaf of the wide ligament. It shows the ureters lateralized and the posterior leaf of the wide ligament. After that, the ureters’ path is shown, the retroperitoneal area is revealed, and the location where the uterine artery leaves the iliac artery is seen. The ureter is gently moved medially to prevent electrical harm, and the region where the uterine artery and ureter meet is exposed. The uterine artery’s ascending branch can be distinguished at the point where it splits off from the hypogastric artery. At this point, the uterine arteries are either coagulated using a bipolar tissue sealer or tied with titanium clips. One of the three methods will be used to gain access to the uterine artery: anterior, pelvic, or lateral.

##### 2.3.1.1 In the lateral approach

We start the dissection for vessel ligation from the anterior leaf of the broad ligament. The triangle enclosed by the round ligament, external iliac artery, and infundibulopelvic ligament is opened. The areolar space is dissected, and the origin of the uterine artery from the internal iliac is identified. It is important at this point to also identify the ureter and its relation to the uterine artery to avoid inadvertent ligation. The uterine artery is isolated from the surrounding structures and ligated (24).

##### 2.3.1.2 In the anterior approach

The anterior leaf of the broad ligament is opened, and the uterine artery is clipped lateral to its crossing over the ureter under the guidance of the obliterated umbilical ligament (25).

##### 2.3.1.3 In the pelvic approach

The posterior leaf of the broad ligament is opened, and the uterine artery is identified at the bifurcation from the hypogastric artery under the guidance of the obliterated umbilical ligament and then ligated (26).

The uterine artery is coagulated at its origin from the internal iliac artery. This is followed by the coagulation and section of either ovarian (infundibulopelvic ligament) if adnexectomy is performed or utero-ovarian vessels.

#### 2.3.2 Group 2 (conventional TLH)

The conventional TLH technique involved the division of the corneal pedicles and securing the uterine pedicles. Preoperative preparation of the bowel was not routinely performed to improve recovery in patients. Antibiotic prophylaxis with third-generation cephalosporin and metronidazole was given 1 h preoperatively. Obese patients received subcutaneous low molecular weight heparin and compression devices after surgery. Under general anesthesia, patients were placed in a Lloyd Davis position. The cervix was held with a vulsellum at the anterior lip and dilated to 10 Hegar. The appropriate intrauterine element was selected based on the uterocervical length and vaginal delineated cup width. A Veress needle was inserted into the umbilicus, and the abdomen was insufflated with carbon dioxide (CO2). Trocars were placed under direct vision, and the abdomen was surveyed before starting the procedure. Vessel sealing, thermostable, and bipolar electric currents were used for securing the vascular pedicles. Monopolar cutting energy was used for cutting the vaginal fornices. The primary outcome was intraoperative blood loss, and secondary outcomes included operative time (from insufflation to skin suturing), intraoperative and postoperative complications, postoperative analgesic needs, and hospital stay.

##### 2.3.2.1 In both groups

The vaginal vault is identified and cut laparoscopically using a monopolar hook over the manipulator cup and a bipolar grasper for hemostasis until the specimen is detached completely.

The total blood loss is calculated from the suction apparatus. If irrigation is used, it is considered and subtracted. Blood Hg is measured 6 h postoperative and is compared to pre-operative Hg.

Operative time is calculated from the insertion of the Veress needle for the establishment of the pneumoperitoneum to the time of suturing port sites. The time required for anesthesia and patient positioning, as well as the time needed for recovery, is not calculated in operative time but is considered for assessment of anesthetic complications, e.g., delayed recovery.

### 2.4 Sample size calculation

The sample size was determined based on estimated blood loss, with approximately 70.96 ± 18.33 mL for group A; and 43.08 ± 5.67 mL for group B. TLH was performed by ligating both uterine arteries at the beginning of the procedure, as reported in a previous publication by Poojari et al. (20). In order to use a t-test for independent samples with 80% statistical power and reject the null hypothesis at a significance level of *α* = 0.05, each group had to have at least 22 participants. In order to account for the dropout rate, we added 30 individuals to each group for the study. MedCalc^®^ Statistical Software version 19.5.3 (MedCalc Software Ltd., Ostend, Belgium; https://www.medcalc.org; 2020) was used to calculate the sample size.

### 2.5 Preoperative preparation

Antibiotic prophylaxis with third-generation cephalosporin and metronidazole is given to all patients 1 h preoperatively. Compression devices and subcutaneous low molecular weight heparin postoperatively are given to obese patients for prophylaxis of possible thromboembolic episodes.

### 2.6 Anesthesia and positioning

Under general anesthesia, patients are placed in a modified lithotomy position.

### 2.7 Insertion of uterine manipulator “Mangeshker Uterine Manipulator (MUM)”

The cervix is held with a vulsellum at the anterior lip and dilated to 10 Hegar. The uterine length is determined with a uterine sound, and the appropriate intrauterine element (IUE) is selected. The correct IUE is chosen to reduce the risk of uterine perforation and trauma. The Vaginal Delineating Cup (VDC) is selected depending on the width of the vagina and the diameter of the cervix (27).

### 2.8 Abdominal entry and trocar positioning

A Veress needle is inserted into the umbilicus, and the abdomen is to be insufflated with carbon dioxide at an initial pressure of 20 mmHg and maintained at 15 mmHg. A 10-mm trocar is inserted blindly, and a 10-mm 0° telescope is introduced through this port; however, a 10-mm 30° telescope was used in some cases. A complete survey of the abdomen was performed to rule out any visceral injury at the time of entry, then the uterus and the adnexa were visualized.

Other trocars are placed under direct vision. Two trocars are placed lateral to the rectus abdominis muscles, 2 cm above and 2 cm medial to the anterior superior iliac spine; a 5-mm trocar is placed on the right and a 10-mm trocar on the left. In addition, a fourth 5-mm trocar is placed suprapubicly at the midline (28). The entire abdomen is surveyed before starting the procedure. The size of the uterus, presence of myomas, adnexa, and course of the ureters are visualized.

### 2.9 Energy systems used

Vessel sealing (ThermoStapler) with a reusable laparoscopic handle is used for securing and coagulating vascular pedicles, which are the uterine, ovarian, or utero-ovarian vessels.

### 2.10 Postoperative care

Follow-up on vital signs and urine output hourly for the first 6 h. The catheter is removed after 6 h, and a liquid diet is started after peristalsis is established. Postoperative analgesics in the form of intravenous non-steroidal anti-inflammatory drugs (NSAIDs) are given according to the patient’s pain score. Early movement is encouraged. Patients are discharged the following day if there are no complications and called for follow-up after 7 days.

### 2.11 Study outcomes (items evaluated)

1. Intraoperative blood loss
2. Operative time
3. Intra and postoperative complications
  - Major surgical complications
    - Urinary tract injury (bladder or ureter)
    - Vascular injury
    - Intestinal injury
  - Other complications
    - Delayed recovery and other anesthetic complications
    - Wound infection
    - Paralytic ileus
    - Febrile complications
    - Urinary tract infection
    - Vaginal bleeding
    - Vault dehiscence
    - Port site hernias
4. Postoperative analgesic needs
5. Hospital stays

### 2.12 Statistical analysis

Statistical analysis was conducted using IBM SPSS Statistics^®^ 28 for Windows. The Shapiro–Wilk test was used to assess the normality of the data. While categorical variables were represented by counts and percentages, continuous variables were represented by means and standard deviations. Paired and unpaired t-tests were used for comparison within and between groups, respectively, for continuous variables. The Mann–Whitney U-test and Wilcoxon test were used for comparison within and between groups, respectively, for non-parametric data. For categorical variables, Fisher’s exact test and the chi-square test were used. Pearson’s correlation was used to measure the association between parameters. Statistical significance was established at *p* < 0.05.

## 3 Results

### 3.1 Analysis of baseline demographic data

This prospective randomized clinical study included 60 patients, divided equally into 2 groups: 30 patients undergoing total laparoscopic hysterectomy with bilateral uterine artery ligation (BUAL) at its origin, and 30 patients undergoing conventional total laparoscopic hysterectomy (CTLH). Table 1 shows that there were no significant differences between the BUAL and CTLH groups regarding age, ALT, AST, serum creatinine, and BMI, indicating that both groups were well-matched demographically at the study’s outset.

**Table 1:** Comparison between the two studied groups according to the demographic data.

Character	BUAL (*n* = 30)	CTLH (*n* = 30)	t	*p*
Age (years)				
Min. – Max.	38.0–54.0	38.0–53.0	1.600	0.115
Mean ± SD.	47.60 ± 4.06	45.87 ± 4.32		
Median (IQR)	48.0 (46.0–51.0)	46 (42.0–50.0)		
BMI (kg/m^2^)				
Min. – Max.	22.0–42.0	22.0–35.0	0.760	0.450
Mean ± SD.	28.50 ± 4.26	27.73 ± 3.51		
Median (IQR)	28 (26.0–31.0)	28 (25.0–30.0)		
ALT (IU/L)	27.24 ± 4.25	29.24 ± 5.25	0.847	0.263
AST (IU/L)	31.25 ± 3.54	28.24 ± 6.23	0.485	0.632
Sr.Cr (mg/dl)	0.98 ± 0.54	1.02 ± 0.24	0.635	0.247
RBCs (10^6^)	4.57 ± 0.623	4.63 ± 0.651	0.327	0.741

Data are presented as mean, standard deviation, median, and interquartile range. BMI, body mass index, ALT, alanine aminotransferase; AST, aspartate aminotransferase; Sr.Cr, serum creatinine, RBCs, red blood cells, BUAL, bilateral uterine artery ligation, CTLH, conventional total laparoscopic hysterectomy. t: Student’s t-test, *p*: *p*-value for comparing between the studied groups. Significance is indicated by a *p*-value less than 0.05.

### 3.2 Comparison between the two studied groups according to the mode of delivery, parity, and indication of operation

Tables 2, 3 compare parity and mode of delivery between the two groups, showing no significant differences in their previous surgical and obstetric history.

**Table 2:** Comparison between the two studied groups according to parity.

Parity	BUAL (*n* = 30)		CTLH (*n* = 30)		^FE^ *p*
	No.	%	No.	%	
Nullipara	3	10.0	1	3.3	0.612
Multipara	27	90.0	29	96.7	

Data are presented as numbers and percentages. BUAL, bilateral uterine artery ligation and CTLH, conventional total laparoscopic hysterectomy. FE: Fisher’s exact test. *p*: *p*-value for comparing between the studied groups.

**Table 3:** Comparison between the two studied groups according to the mode of delivery.

Mode of delivery	BUAL (*n* = 27)		CTLH (*n* = 29)		*P*
	No.	%	No.	%	
NVD	22	81.5	23	79.3	0.838
CS	5	18.5	6	20.7	

Data are presented as numbers and percentages. BUAL, bilateral uterine artery ligation, CTLH, conventional total laparoscopic hysterectomy, CS, cesarean section, NVD, normal vaginal delivery. *p*: *p*-value for comparing between the studied groups using Fisher’s exact test.

Table 4 shows no statistically significant differences between the groups regarding operative indications. Abnormal uterine bleeding (AUB) was the most common indication for surgery in both groups, followed by leiomyoma and adenomyosis.

**Table 4:** Comparison between the two studied groups according to the indication of operation.

Indication of operation	BUAL (*n* = 30)		CTLH (*n* = 30)		*p*
	No.	%	No.	%	
AUB	12	40.0	10	33.3	0.592
Adenomyosis	5	16.7	7	23.3	0.519
Prolapse	3	10.0	0	0.0	^FE^p = 0.237
Fibroid	9	30.0	11	36.7	0.584
Endometrial hyperplasia with atypia	5	16.7	3	10.0	^FE^p = 0.706
Endometrial hyperplasia without atypia	1	3.3	3	10.0	^FE^p = 0.612

Data are presented as numbers and percentages. AUB, abnormal uterine bleeding, BUAL, bilateral uterine artery ligation, CTLH, conventional total laparoscopic hysterectomy. FE: Fisher’s exact test. *p*: *p*-value for comparing between the studied groups.

### 3.3 Comparison between the two studied groups according to the operative parameters

Table 5 shows that the mean operating time was slightly longer in the BUAL group than in the CTLH group. The amount of blood loss in the BUAL group was significantly lower than in the CTLH group (*p* < 0.05). The change in hemoglobin levels (the difference between pre-operative and postoperative hemoglobin) was not significantly different between the groups.

**Table 5:** Comparison between the two studied groups according to operative parameters.

Operative parameters	BUAL (*n* = 30)	CTLH (*n* = 30)	t	*p*
Operating time (min)				
Min. – Max.	145.0–205.0	130.0–205.0	1.910	0.061
Mean ± SD.	169.33 ± 15.85	160.50 ± 19.75		
Blood loss (ml)				
Min. – Max.	70.0–150.0	70.0–220.0	2.823^*^	0.007^*^
Mean ± SD.	103.7 ± 23.27	128.7 ± 42.57		
Hemoglobin				
Preoperative	10.90 ± 0.64	10.70 ± 0.53	1.344	0.184
Postoperative	10.35 ± 0.51	10.09 ± 0.46	2.069^*^	0.043^*^
Change (g %)	0.55 ± 0.30	0.60 ± 0.24	0.813	0.420

Data are presented as mean and standard deviation. BUAL, bilateral uterine artery ligation, and CTLH, conventional total laparoscopic hysterectomy. t: Student’s t-test, *p*: *p*-value for comparing between the studied groups. Significance is indicated by a *P*-value less than 0.05. p_1_: *p*-value for comparing between pre and post in each group, *: statistical significance is indicated by a *p*-value less than 0.05.

Table 6 shows no significant differences in the incidence of postoperative complications between the BUAL and CTLH groups.

**Table 6:** Comparison between the two studied groups according to post-operation complications.

Post-operation complications	BUAL (*n* = 30)		CTLH (*n* = 30)		χ^2^	*p*
	No.	%	No.	%		
No	23	76.7	23	76.7	0.000	1.000
**Yes**	**7**	**23.3**	**7**	**23.3**		
Wound infection	2	33.3	4	66.7	1.333	^FE^*p* = 0.567
Fever	4	66.7	2	33.3		
Urinary tract injury	0	0.0	0	0.0	–	–
Bowel injury	0	0.0	0	0.0	–	–
Vascular injury	0	0.0	0	0.0	–	–
Delayed recovery	1	3.3	1	3.3	0.00	1.000
Paralytic ileus	0	0.0	0	0.0	–	–
UTI	0	0.0	0	0.0	–	–
Vaginal bleeding	0	0.0	0	0.0	–	–
Port site hernia	0	0.0	0	0.0	–	–

Data are presented as numbers and percentages. UTI, urinary tract infection, BUAL, bilateral uterine artery ligation, CTLH, conventional total laparoscopic hysterectomy. χ^2^: chi-square test. FE: Fisher’s exact test. *p*: *p*-value for comparing between the studied groups.

Table 7 shows that postoperative analgesic requirements and hospital stay were not significantly different between the two groups.

**Table 7:** Comparison between the two studied groups according to postoperative analgesic need (number of times) and hospital stay (days).

Character	BUAL (*n* = 30)	CTLH (*n* = 30)	U	*p*
Postoperative analgesic need (number of times)				
Min. – Max.	1.0–5.0	1.0–5.0	413.0	0.566
Mean ± SD.	2.43 ± 1.17	2.27 ± 1.11		
Median (IQR)	2.0 (2.0–3.0)	2.n (2.0–3.0)		
Hospital stays (days)				
Min. – Max.	1.0–3.0	1.0–2.0	365.0	0.146
Mean ± SD.	1.57 ± 0.63	1.33 ± 0.48		
Median (IQR)	1.50 (1.0–2.0)	1.0 (1.0–2.0)		

Data are presented as mean, standard deviation, median, and interquartile ranges. IQR, interquartile range, BUAL, bilateral uterine artery ligation, CTLH, conventional total laparoscopic hysterectomy. U: Mann–Whitney U-test. *p*: *p*-value for comparing between the studied groups.

Table 8 compares the different approaches to uterine artery ligation from its origin in the BUAL group. The posterior approach (mean time 16.3 ± 3.9 min) was found to be quicker than both the anterior (mean time 23.33 ± 9.8 min) and lateral (mean time 17.14 ± 2.3 min) approaches, suggesting that the posterior approach is easier and more efficient.

**Table 8:** Comparison between the different approaches to uterine artery ligation from its origin according to time.

Character	Side approach			H	*p*
	Anterior (*n* = 6)	Lateral (*n* = 14)	Posterior (*n* = 40)		
Side time (min.)					
Min. – Max.	15–40	15–20	10–30	5.491	0.064
Mean ± SD.	23.33 ± 9.8	17.14 ± 2.3	16.3 ± 3.9		
Median (IQR)	20 (15–30)	15 (15–20)	15 (15–20)		

Data are presented as mean, standard deviation, median, and interquartile ranges. H: H for Kruskal–Wallis test.

### 3.4 Correlation analysis between the measured parameters

**Figure 2:**
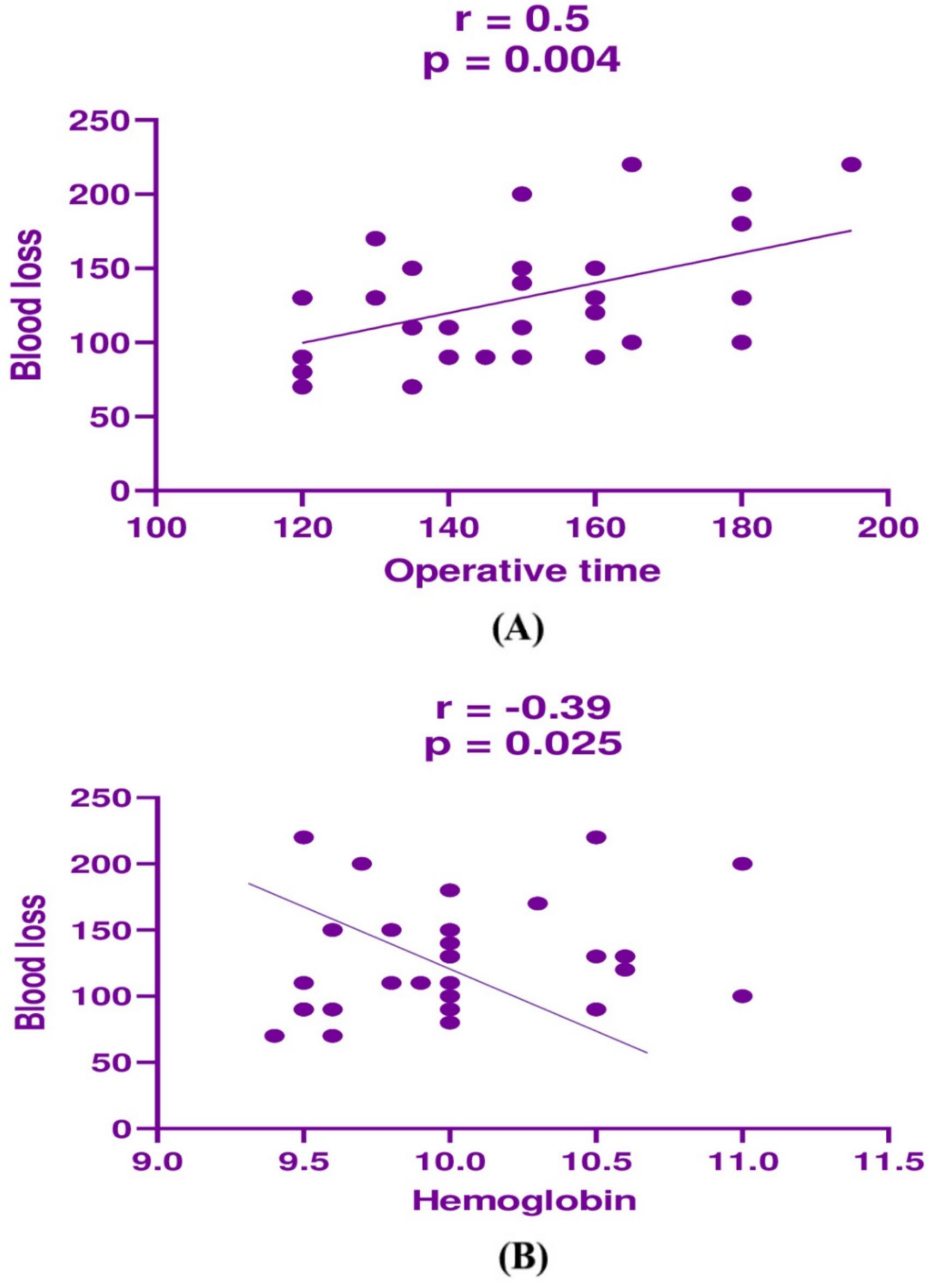
Correlation analysis between **(A)** operative time and blood loss in the BUAL group and **(B)** between operative time and hemoglobin in the BUAL group.

A significant positive correlation was observed between operative time and blood loss in the BUAL group (*p* = 0.004, r = 0.5, Figure 2A), and a significant negative correlation was observed between operative time and hemoglobin level (*p* = 0.025, r = −0.39, Figure 2B).

## 4 Discussion

Hysterectomy techniques have undergone significant advancements over the past few decades, transitioning from traditional open surgeries to minimally invasive approaches that prioritize patient safety, faster recovery, and reduced morbidity (29). Historically, total abdominal hysterectomy (TAH) was the standard approach, offering direct visualization and accessibility. However, the shift toward minimally invasive techniques has led to a marked reduction in surgical trauma, postoperative pain, and hospital stays (30). The introduction of laparoscopic hysterectomy (LH) in the 1980s revolutionized gynecological surgery, providing a viable alternative to open surgery. Compared to TAH, LH demonstrated clear advantages, including reduced intraoperative blood loss, fewer wound complications, and a quicker return to daily activities. In recent years, the development of total laparoscopic hysterectomy (TLH) has further refined minimally invasive gynecological procedures (31). TLH allows complete detachment and removal of the uterus using laparoscopic techniques, leading to enhanced surgical precision and better patient outcomes. It is suggested that TLH is the future of hysterectomy in the 21st century (32). The exponential growth in the past decades in the field of endoscopic surgery has allowed a marked diffusion of TLH. Minimally invasive approaches have been applied with success to an increasing number of gynecological procedures. In the United States of America, robotics has spread to many centers, setting the standard for minimally invasive gynecological procedures (33). In contrast, in Europe, TLH is much more widely distributed, being the main alternative to TAH (34).

The evolution of laparoscopic instruments and energy devices, such as harmonic scalpels and vessel-sealing technologies, has played a crucial role in improving surgical efficiency and reducing complications (35). Studies comparing different hysterectomy techniques suggest that TLH, when performed by experienced surgeons, is associated with lower overall complication rates and shorter recovery times than abdominal hysterectomy (36). Additionally, the adoption of robotic-assisted hysterectomy has provided further advancements in minimally invasive surgery, offering superior dexterity, 3D visualization, and enhanced ergonomics for surgeons (37). Over the last decade, continuous refinements in laparoscopic techniques have aimed to minimize complications, particularly ureteral and bladder injuries. Enhanced surgical training programs and standardized procedural protocols have contributed to improved patient safety (37).

TLH offers several benefits over TAH, including shorter hospital stays, less blood loss, and lower rates of surgical infections and ileus. Patients avoid a painful abdominal incision, allowing for quicker recovery (38). It is well known that laparoscopy, when compared to open surgery, offers advantages to both the patient and the surgeon. However, when compared to abdominal or vaginal hysterectomy, it is still associated with a higher incidence of major intra-and postoperative complications (39–42). According to the literature, the incidence of ureteral injuries in total abdominal hysterectomy ranges from 0.04 to 0.4% (43), whereas in LH, it ranges from 0.65 to 1.39% (39). The true rate of urinary tract injuries is hard to determine due to various interfering factors during surgery. Ribeiro et al. reported a 3.4% incidence of ureteral injuries with cystoscopy. In a study by Ribeiro et al. (44), cystoscopy performed at the time of the procedure revealed a 3.4% incidence of ureteral injuries. However, this high percentage of ureteral damage was supposed by other authors to be correlated with patient selection (many patients with endometriosis and with a large uterus) and the technique of uterine artery closure (suture rather than bipolar electrocoagulation) (45).

The incidence of bladder injury ranges from 0.2 to 1.8%, with recent studies indicating that these rates are similar to those of patients undergoing abdominal hysterectomy (46). Bladder injury appears to be significantly associated with previous laparotomy, adhesiolysis, and, in particular, previous cesarean sections (46). Ureteral injury is strongly associated with thermal spread from coagulation devices or with suture ligation during uterine artery occlusion and vaginal cuff closure (47). Although bladder damage is easier to recognize and repair, ureteral injury is more insidious, and many efforts have been made to reduce this complication. Some authors suggest that the dissection and isolation of the ureter during surgery is necessary to prevent ureteral injury, but this technique extends the operative time, increases the risk of bleeding, and requires a long learning curve (48, 49).

The most widespread technique for TLH involves intrafascial dissection of the vascular pedicles and the use of a uterine manipulator for the mobilization of the uterus and the cervix. In TLH procedures, the securing of the uterine vessels is performed close to the uterus and medially to the ureters, repeating the steps of a conventional TAH (34, 38, 50). Dissection or coagulation of the uterine vessels may be sometimes difficult, however, due to a variety of anatomical variations such as intra-ligamentary fibroids, endometriosis, or pelvic inflammatory conditions. In addition, extensive coagulation increases the risk of ureteral lesions (39, 51, 52).

Several options for securing the pedicles are available to the laparoscopic surgeon, including bipolar diathermy, harmonic ultracision, vessel-sealing instruments, laparoscopic suturing, or staples (53). Complications such as hemorrhage and bladder and ureteral injuries are related to the method of securing the vascular pedicles. The vascular supply of the uterus is derived principally from the uterine and ovarian arteries. Since the majority of the blood supply to the uterus is delivered through the uterine arteries, transient uterine ischemia can occur after uterine artery ligation (UAL) (54). Bilateral uterine vessel ligation is an efficient method to obliterate the blood flow to the uterus. To reduce the total blood loss and the duration of surgery, in this study, we secured the uterine arteries as the first step before tackling the other pedicles.

Bilateral uterine vessel ligation effectively stops uterine blood flow, reducing total blood loss and surgery duration when done at the beginning of TLH (54). Studies have shown that securing uterine arteries first in TLH (BUAL from its origin) results in lower blood loss compared to conventional TLH (CTLH). Sinha et al. found a median blood loss of 50 mL and surgery duration of 60 min in the BUAL group versus 60 mL and 70 min in the CTLH group (55). Poojari et al. reported blood loss of 43 mL and surgery time of 60.77 min in the BUAL group compared to 70 mL and 71.35 min in the CTLH group (20). Kale et al. found longer surgery times (99.16 min) and higher blood loss (109.38 mL) in the CTLH group compared to the BUAL group (63.27 min and 47.50 mL) (56). Our study supports these findings, showing significantly reduced blood loss in the BUAL group compared to the CTLH group (*p* < 0.05). However, the mean operative time (from insufflation to skin suturing) was slightly lower in the CTLH group (160.50 ± 19.75 min) compared to the BUAL group (169.33 ± 15.85 min), likely due to the time required for uterine artery dissection and ligation. The longer operative times in our study (130 to 205 min) compared to other studies (60 to 90 min) may be attributed to more time spent on exploration, uterine manipulation, dissection, vessel securing, and intracorporeal sutures.

Our study showed that the average blood loss during the procedure is considerably reduced if the uterine vessels are primarily secured. Mean blood loss of 103.7 ± 23.27 mL versus 128.7 ± 42.57 mL when CTLH was done. The difference was statistically significant (*p*-value < 0.5). Therefore, according to Sinha et al., Poojari et al., Kale et al., and our study, blood loss is less in the BUAL group than in the CTLH group.

In our study, the mean operative time was a bit lower in the CTLH group (160.50 ± 19.75) compared to (169.33 ± 15.85) in the BUAL group, although this difference was not statistically significant (*p*-value = 0.061). We believe that the cause is the time taken to dissect and ligate the uterine artery from its origin.

We observe that in our study, the mean operative time in both groups (range: 130 min to 205 min) is more than that observed in other studies, approximately 60 to 90 min. We believe that the longer operative in our study belongs to more time being consumed for exploration, uterine manipulation, dissection, securing vessels on multiple steps, and intracorporeal sutures of the vaginal vault. As evidenced by the improvement in operative time throughout the study with the longer operative time (+3 h) in the first few cases compared to less than 2 h duration at the end of the study, there is marked progression in the learning curve of our team.

In our study, except for some minor complications, none of the 60 cases included in our study experienced major surgical complications such as bladder, ureteral injury, or bowel injury. Additionally, no cases needed a blood transfusion.

Sinha et al. (19) reported no major complications in their study. One patient in the study group had a secondary hemorrhage 3 weeks later, and the vaginal vault was resutured. In control group 2, patients had blood loss of more than 1,500 mL (uterus weight 1,000 g) and required 4 units of packed cell transfusions. One patient in the control group with a previous cesarean section had a bladder wall rent, and this was sutured laparoscopically using 3–0 delayed absorbable sutures. The urinary catheter was removed after 1 week, and the patient had an uneventful postoperative period. Furthermore, Poojari et al. (20) reported that no major complications had occurred. Only one patient in the control group with multiple fibroids and the previous two lower segment cesarean sections (LSCSs) had bladder injury, was detected postoperatively and was treated conservatively with catheterization for 2 weeks. Kale et al. (56) in their study reported no complications in either group. The complications after laparoscopic hysterectomy are influenced by the surgeon’s experience (57), after a decade of surgical experience, the overall complication rate during total LH was significantly reduced from 4.5% (LH between 1994 and 2001) to 1.5% (LH between 2001 and 2007). It appears that at least 30 procedures are necessary to achieve a significant decrease in bladder and ureter injury (58).

Our study has some limitations. The number of cases is smaller than in other studies, and the study was not multicentric.

In summary, while the BUAL group experienced significantly less blood loss compared to the CTLH group, there were no significant differences between the groups in terms of operating time, postoperative complications, analgesic requirements, or hospital stay. The posterior approach to uterine artery ligation was the most time-efficient.

## 5 Conclusion

Securing the bilateral uterine arteries at their origin prior to TLH significantly reduces blood loss without a significant increase in operative time or perioperative morbidity. As the expertise of the surgeon increases, the duration of the procedure reduces considerably.

## Data Availability

The raw data supporting the conclusions of this article will be made available by the authors, without undue reservation.
